# Influenza virus N-linked glycosylation and innate immunity

**DOI:** 10.1042/BSR20171505

**Published:** 2019-01-08

**Authors:** Ian A. York, James Stevens, Irina V. Alymova

**Affiliations:** Influenza Division, National Center for Immunization and Respiratory Diseases, Centers for Disease Control and Prevention, Atlanta, GA, 30329, U.S.A.

**Keywords:** influenza, innate, immunity

## Abstract

Influenza viruses cause seasonal epidemics and sporadic pandemics in humans. The virus’s ability to change its antigenic nature through mutation and recombination, and the difficulty in developing highly effective universal vaccines against it, make it a serious global public health challenge. Influenza virus’s surface glycoproteins, hemagglutinin and neuraminidase, are all modified by the host cell’s N-linked glycosylation pathways. Host innate immune responses are the first line of defense against infection, and glycosylation of these major antigens plays an important role in the generation of host innate responses toward the virus. Here, we review the principal findings in the analytical techniques used to study influenza N-linked glycosylation, the evolutionary dynamics of N-linked glycosylation in seasonal versus pandemic and zoonotic strains, its role in host innate immune responses, and the prospects for lectin-based therapies. As the efficiency of innate immune responses is a critical determinant of disease severity and adaptive immunity, the study of influenza glycobiology is of clinical as well as research interest.

## Introduction

Influenza viruses have caused epidemic and pandemic disease in humans for hundreds of years [1], but the disease remains difficult to predict and prevent. Three types of influenza viruses infect humans: A, B, and C. While wild waterfowl and other birds are the primary reservoirs of influenza A virus (IAV) [2], humans are the primary host and reservoir of influenza B and C viruses (IBV and ICV, respectively) [3,4]. In addition to the primary reservoirs, IAV can infect many other species, including domestic species such as pigs, horses, dogs, and cats [2,5]. IBV and ICV are less broadly infectious but have been shown to infect seals [6], pigs, and dogs [7,8]. There are therefore many sources of influenza virus exposed to humans.

Among the three influenza types, IAV is the greatest public health concern because of its ability to cause not only widespread seasonal epidemics, but also pandemics originating from zoonotic infections [9–13]. IBV has not yet been associated with pandemics but causes seasonal epidemics and is responsible for ∼25% of human seasonal influenza infections [14,15]. Together, seasonal epidemics of IAV and IBV can cause up to 5 million infections and 500,000 deaths annually [16]. Type C virus typically causes a mild respiratory illness [17] and in contrast with IAV and IBV, it is not included in the composition of seasonal influenza vaccines. As it is of less public health concern, ICV has been less studied than IAV and IBV. Therefore, this review will focus on IAV and IBV only.

## Influenza glycoproteins

**Figure 1:**
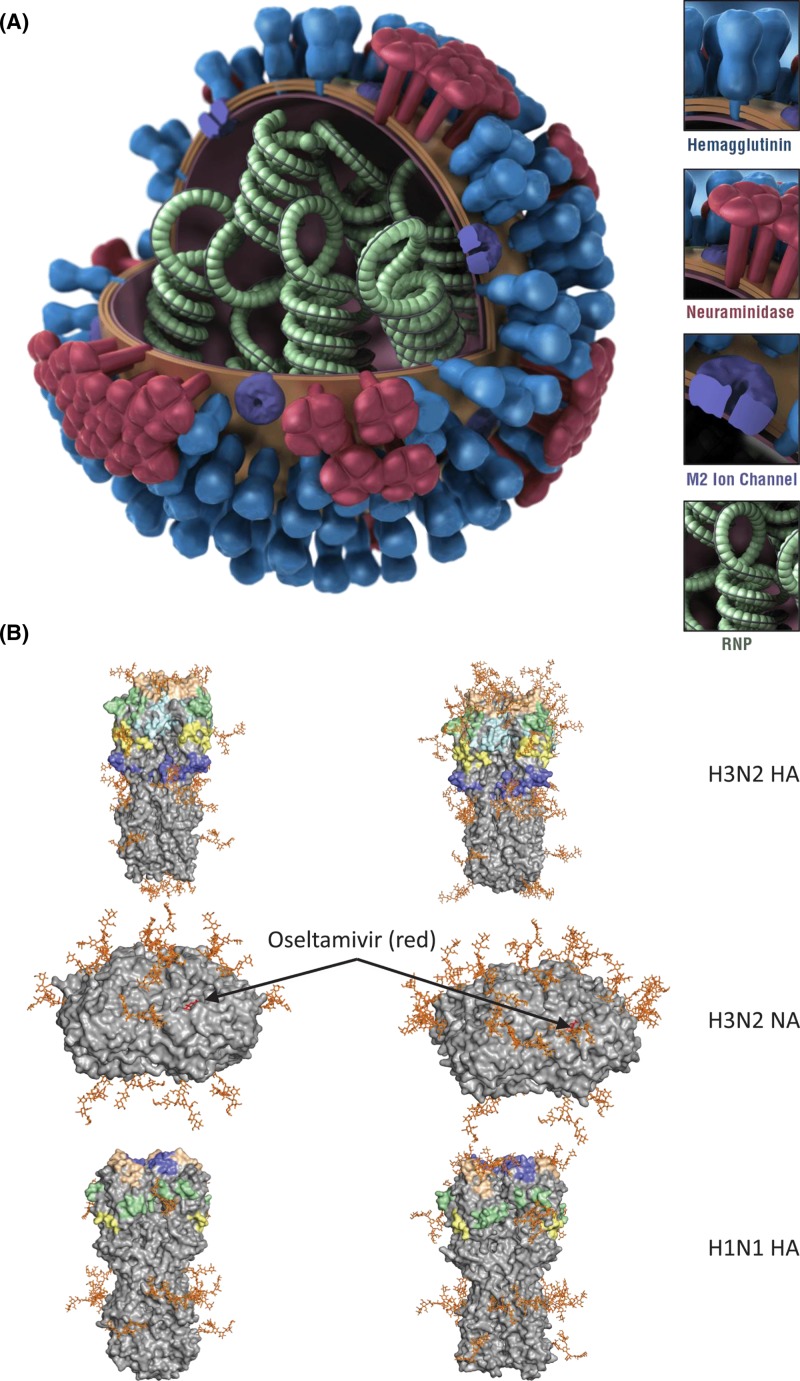
Influenza virus surface glycoproteins (**A**) Schematic diagram of an influenza virion, showing HA and NA (provided by CDC Influenza Division/Douglas Jordan/Dan Higgins). (**B**) Structure of HA (top and bottom rows) and NA (middle row) showing NLG sites for early (left columns) and recent (right column) isolates of A(H3N2) and A(H1N1) IAV. ‘Oseltamivir’ (in red) indicates the NA active site. Early A(H3N2) isolate: A/Hong Kong/1/1968(H3N2). Recent A(H3N2) isolate: A/Singapore/INFIMH-16-0019/2016(H3N2). Early A(H1N1) isolate: A/South Carolina/1/1918(H1N1). Recent A(H1N1)isolate: A/Brisbane/59/2007(H1N1).

Influenza viruses belong to the Orthomyxoviridae family and contain a genome consisting of eight negative sense RNA segments. Segments four and six encode two major virus surface glycoproteins, the hemagglutinin (HA), a trimeric lectin, and the neuraminidase (NA), a tetrameric enzyme (Figure 1A). The number of NAs on the viral surface is up to 10-fold less than HAs [18]. The external parts of both proteins consist of a stalk and globular head regions. HA is the virus’s primary receptor-binding protein, binding to specific receptors that possess terminal *N-*acetylneuraminic acid (Neu5Ac or sialic acid [SA]), particularly on epithelial cells and mucins of human respiratory tracts (RT) [19]. SA-independent binding and internalization of influenza virus HA also occur via specific cell-surface proteins on alveolar and recruited monocytes/macrophages, dendritic cells (DCs), and B cells [20–26]. Binding to cell receptors is the first step in the process leading to a productive virus infection. As with HA, NA also has specificity for terminal SA [27]. The primary role of influenza virus NA is to cleave SA residues from virus and cellular glycoproteins at the time of the budding of newly formed virions from the surface to prevent virus-self aggregation and enable its release from infected cells. As well, NA cleaves SA from the mucins of the RT to facilitate viral movement to target cells [28–30].

IAV HA and NA are antigenically variable, with 18 structural variants of IAV HA and 11 variants of NA have been identified [31]. IAV subtypes are named based on their HA and NA combinations. In the 20th and 21st centuries, most IAV-related diseases in humans have been caused by the A(H1N1), A(H2N2), and A(H3N2) subtypes. In 1918, the highly pathogenic pandemic A(H1N1) killed as many as 50–100 million people [32]. In 1957, A(H1N1) was replaced by A(H2N2), and in 1968 the A(H3N2) strain caused a global pandemic associated with more than one million deaths [33]. In 1977 the A(H1N1) virus re-emerged and co-circulated with A(H3N2) until 2009, when the swine-origin A(H1N1) pandemic (A(H1N1)pdm09) IAV replaced the older, antigenically distinct human seasonal A(H1N1) strain. Humans are also sporadically infected with highly and low pathogenic avian influenza A viruses, particularly H5, H9, and H7 HA subtypes. As yet, these viruses do not efficiently spread from person to person.

The surface glycoproteins of IAV, especially HA, are very tolerant of amino acid variations [34], allowing them to accumulate mutations that alter their antigenic nature. As IAV circulates in humans, HA and to a lesser extent NA [34] are therefore able to undergo repeated antigenic changes in response to population immunity. This ‘antigenic drift’ presents a public health challenge because of the need to adjust the composition of seasonal vaccine in most years to provide protection against newly circulating antigenic variants. In contrast with IAV, IBV do not have subtypes of HA and NA. Influenza B viruses were first identified in the 1940s, and in the 1980s evolved into two genetically and antigenically distinct lineages, the B/Victoria/2/87 and the B/Yamagata/16/88 lineages. Co-circulation of both influenza B virus lineages with influenza A(H3N2) and A(H1N1) viruses during seasonal epidemics has prompted the development of quadrivalent vaccines that include strains from both subtypes of IAV and both influenza B lineages.

## Influenza virus glycosylation

As well as simple amino acid changes, antigenic drift often involves changes in influenza virus HA and NA glycosylation patterns that shield or expose particular antigenic sites on these glycoproteins [35]. Host–cell dependent glycosylation is a critical post-translational modification of influenza virus HA and NA. Glycosylation in the HA stalk region is important for protein folding and trafficking and for pH stability [36–38], while the extent of glycosylation near the HA receptor-binding site alters its affinity for SA-containing receptors [39–41]. Glycosylation near the cleavage HA site also modulates virus pathogenicity [42,43]. While the role of glycosylation of NA is less well understood, N-linked glycosylation is important for functional NA [44], and lack of NA glycosylation increases neurovirulence of the A/WSN/33 IAV strain in mice [45].

Glycosylation of HA and NA occurs through attachment of an oligosaccharide molecule to the side-chain amide nitrogen of Asn in the context of the conserved N-linked glycosylation (NLG) sequon Asn-X-Ser/Th, where X may represent any amino acid except Pro [46]. This attachment occurs with the help of glycosyltransferases and oligosaccharyltransferase in the endoplasmic reticulum (ER) and is followed by a complex process of trimming and remodeling of the oligosaccharides by glycosidases and mannosidases during transit through the ER and Golgi. This process results in a glycoprotein whose N-linked oligosaccharides branched structures terminate in mannose (high-mannose glycans), galactose and/or N-acetyl-galactosamine/fucose (complex glycans), or a combination (hybrid glycans) [47,48]. Some influenza virus HA and NA NLG sites may contain predominantly high-mannose glycans as the result of steric hindrance preventing access to the glycosyltransferases [49]. In contrast with cellular proteins, the complex oligosaccharides on influenza virus HA and NA are typically not sialylated, since NA removes the terminal SA [50]. To date, NLG is the only type of glycosylation observed for influenza virus HA and NA: there has been no O-linked (i.e. attachment of a sugar molecule to the hydroxyl group of Ser or Thr on the polypeptide chain) or C-linked (i.e. attachment of a sugar to a carbon group on a Trp side-chain) glycosylation reported for influenza virus.

### Techniques for influenza glycoprotein analysis

The simplest way to predict NLG addition is to identify a sequon, Asn-X-Ser/Thr, within the primary glycoprotein amino acid sequence. However, not all sequons are fully or even partially occupied, for various reasons including amino acid sequence [51–53] and the specific cell type infected by the virus. Amino acid sequence also does not allow accurate prediction of the nature of the glycans added to different sites [54]. Approaches for more detailed analysis of influenza virus NLG have been comprehensively described in a recent review [55]. Briefly, these approaches can be divided into two major groups: those providing information on the nature and relative abundance of the glycoforms present on a glycoprotein, and those that further determine the degree to which each sequon is actually occupied by glycans. The first group of methods requires the initial release of glycans from the glycoproteins (e.g. by using hydrazine or the enzymes PNGase F and PNGase A) followed by mass spectrometry (MS) [56–58] or high-performance liquid chromatography [59,60] for analysis of the released glycoforms. The second group of methods for NLG analysis involves immobilization of glycoproteins (using lectins or periodate-treated glycopeptides linked to hydrazide-activated beads) prior to enzymatic treatment with PNGaseF [61–63]. During the process of glycan release, the Asn-X-Thr/Ser sequence converts to Asp-X-Thr/Ser, resulting in a mass difference. Subsequent MS/MS analysis of the eluted peptides allow identification of the glycosylated sites. Determination of precise glycoforms present at specific glycosites on intact glycoprotein consists of a complex of various techniques and procedures including sophisticated fragmentation by collision-induced dissociation, or high-energy collision dissociation, or electron-transfer dissociation [61,64–67].

Although techniques for comprehensive site-specific characterization of NLG exist and have allowed biochemical verification of the glycans’ presence and their relative abundance at some sites of IAV and IBV glycoproteins [54,68–72], until recently they represented a substantial analytical challenge. In 2018, Cao L. et al. [73] published a much less elaborate protocol for a semiquantitative MS-based method that promises to determine the degree of glycan occupancy at each glycosite and the proportion of N-glycans processed from high-mannose type to complex type. Introduction of this protocol into the ordinary practice of influenza laboratory will improve understanding of glycosylation-associated scientific data.

### Evolutionary dynamics of influenza virus HA and NA N-linked glycosylation

Contemporary human influenza virus strains have circulated in human populations for many decades, undergoing selection for resistance to human immunity as well as enhanced transmission during that period. Glycosylation of influenza virus HA, especially for A(H3N2) viruses, has been shown to have a major impact on antigenicity and is an important factor in antigenic drift [74]. However, increasing glycosylation of HA molecule has also been correlated with decreased virulence, a change in tropism from the lower to the upper RT, and diminished enhancement of secondary bacterial lung infections [75]. This suggests that increasing levels of glycosylation may both enhance influenza immune evasion in the presence of population adaptive immunity, and also reduce viral virulence or replication ability in non-immune individuals.

**Figure 2:**
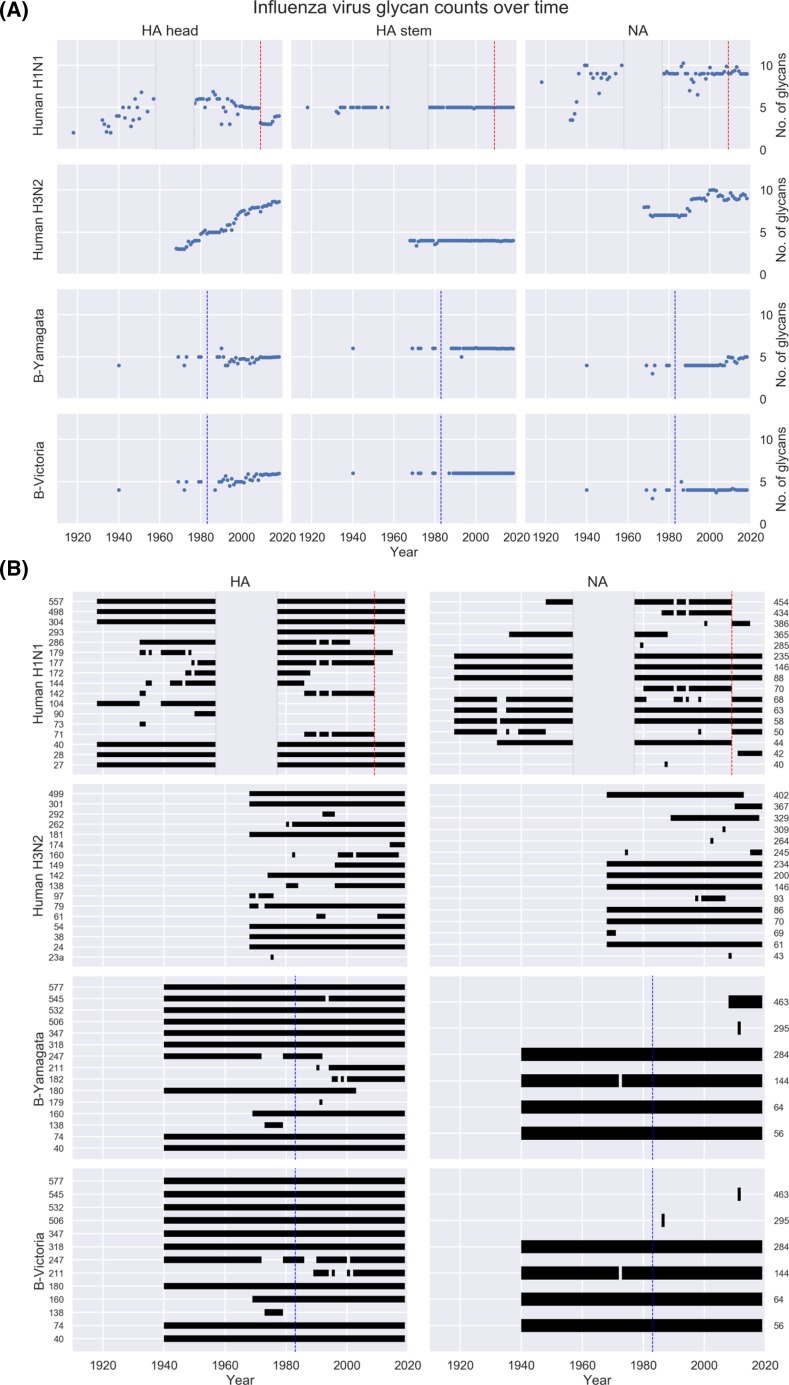
Evolution of influenza virus *N-*linked glycosylation (**A**) Mean number per year of N-linked glycosylation (NLG) sites on the head and HA2 stem regions of HA, and on NA, from human isolates of A(H1N1) and A(H3N2), and B-Yamagata and B-Victoria lineages. (**B**) Residues of HA and NA from human A(H1N1) and A(H3N2) isolates on which more than 10% of isolates contained NLG. HA and NA sequences from human A(H1N1) and A(H3N2) isolates were downloaded from GISAID on September 6, 2018, manually curated to remove duplicates and laboratory variants, and aligned using MAFFT. Sites of potential NLG were identified based on sequence. The residue indicated as ‘23a’ in A(H3N2) represents an insert relative to earlier HA isolates that was present in some viruses isolated in the 1970s. A(H1N1) viruses did not circulate among humans between 1957 and 1976, and those years are not shown. In 2009, indicated by red dashed lines, circulating seasonal A(H1N1) viruses were replaced by a novel pandemic A(H1N1)pdm09 strain. Influenza B viruses split into B-Yamagata and B-Victoria lineages in around 1983 [180]. Influenza B isolates from before 1983 are included in the charts for both lineages, and the date of divergence is indicated with blue dashed lines.

Different influenza strains have shown different patterns of glycosylation in their major surface proteins [76]. The HA of A(H3N2) IAV has shown a strong trend toward increasing numbers of glycans since its introduction into the human population in 1968 (Figure 2A,B). While the glycans on the stalk region have been quite constant for the past 50 years with five NLG sites at residues 24, 38, 54, 301, and 499 (numbering is based on the full-length protein), the globular head has undergone dramatic glycosylation modifications resulting in the loss and, more often, in the gain of glycosylation. The number of NLG sites on the influenza virus HA globular head has increased from two (at residues 97 and 181; site 97 was lost around 1975) on pandemic A(H3N2) IAV to up to nine (at residues 79, 97, 138, 142, 149, 160, 174, 181, and 262) on contemporary seasonal strains. The only new NLG site that has been acquired outside of the globular head was at residue 61, located between stalk and head regions.

The 1918 pandemic A(H1N1) IAV HA had six NLG sites (at residues 27, 28, 40, 304, 498, and 557) within the stalk region and only one (at residue 104) on the globular head of HA (Figure 2A,B). As with A(H3N2), the NLG sites on the HA stalk region of A(H1N1) IAV have been highly conserved, and subsequent glycan losses and gains have occurred within the globular head only. Like A(H3N2), A(H1N1) IAV showed a trend toward increasing glycan number on the globular head until its elimination in 1957 (Figure 2A), but after re-emerging in 1977 the number of glycans remained roughly constant until the virus was replaced by the 2009 A(H1N1)pdm09 pandemic virus (indicated with red dashed lines in Figure 2). This strain had evolved within swine for many decades and was antigenically and genetically more similar to very early human A(H1N1) strains than to the human seasonal A(H1N1) strains that it replaced. The number of NLG sites on human IBV HA has remained fairly constant at 10–12 sites since the split of Yamagata and Victoria lineages (Figure 2A,B) (indicated with blue dashed lines in Figure 2).

Changes in NA glycosylation have been less dramatic than in the HA. The 1918 pandemic A(H1N1) IAV NA had four NLG sites within the stalk region (at residues 50, 58, 63, and 68) and three within the globular head (at residues 88, 146, and 235) (Figure 2A,B). While the stalk region of human IAV N1 maintained a roughly constant number of NLG sites, with sites 50 and 68 being replaced by those at residues 44 and 70, the globular head transiently added NLG at sites 365, 434, and 454. Similarly, the NA from A(H3N2) IAV and IBV have not shown obvious trends for increasing glycan count (Figure 2A,B).

## Influenza glycosylation and innate immune responses

As well as modulating recognition of glycoproteins by the adaptive immune system, glycans on influenza viruses are also important for interactions with the innate immune response. Innate responses are the first line of host defense against viral infection. This system relies on recognition of pathogen-associated molecular properties (PAMPs) that distinguish pathogen molecules from those of the host. For example, terminal mannose residues are features of influenza virus HA and NA, but are not typical of human glycoproteins. When such PAMPs are detected, host pattern recognition receptors (PRRs) are activated, leading to downstream effects that limit pathogen replication, recruit more innate immune cells, and induce and amplify adaptive immune responses, as outlined in detail in a recent review [77]. Although many antiviral PRRs are intracellular, a number of cell-surface and soluble molecules act as antiviral PRRs, and some of these are lectins (proteins containing carbohydrate or fibrinogen recognition domains [CRD and FBG, respectively]) that generate an antiviral effect through binding to either mannose, fucose or galactose derivatives on influenza virus HA and NA [78–83]. The presence and nature of glycans on influenza virus glycoproteins therefore influence the innate immune response to these pathogens.

### C-type lectins

Lectins that require calcium for binding are called C-type lectins [81,84]. These lectins can be present in serum and respiratory secretions as soluble molecules (collectins) or expressed on the surface of innate immune cells (endocytic lectins). The structure of a typical collectin includes an N-terminal cysteine-rich domain, a collagen-like domain (CLD), a neck region, and a CRD responsible for ligand binding [85]. Collectins include surfactant protein D (SP-D) and mannose-binding lectin (MBL), both of which have anti-influenza activity. Endocytic lectins implicated in anti-influenza activity include macrophage mannose receptor (MMR), macrophage galactose-type lectin (MGL), and DCs specific intercellular adhesion molecule-3-grabbing non-integrin (DC-SIGN).

#### Soluble C-type lectins (collectins)

SP-D is well conserved among mammals. It is synthesized by type II pneumocytes and is mainly present in pulmonary surfactant (the lipoprotein substance of the air–liquid interface in the alveoli of the lungs) of mammals including mice, ferrets, and humans [84]. It is also present in tear fluid of mice and humans and probably other mammals [86,87]. SP-D monomers (∼130 kDa each) assemble into a 520 kDa tetrameric form, and further oligomerization of up to eight tetramers can occur, depending on species [88].

MBL is a complement protein that is produced and secreted by liver hepatocytes, and it is normally detected in serum [84]. MBL monomers of ∼32 kDa oligomerize into trimeric to hexameric structures, with trimeric and tetrameric variants being the most usual physiological forms [89]. MBL is highly polymorphic in humans [90,91] resulting in variations in MBL serum levels [92], with ∼10–30% of the global human population being MBL deficient [93]. Variations in MBL levels may contribute to individual responses and may partially explain inconsistent conclusions on the MBL role in infections. SP-D and MBL both preferentially bind mannose over the galactose-type sugars found on influenza virus HA and NA [83].

Both SP-D and MBL have been shown to protect against IAV infection in cell and animal models. SP-D has also shown the ability to neutralize IBV in tissue culture cells [94]. While the SP-D is recognized as a major component of the innate immune response to influenza, the role of MBL in protection against influenza is not as clear [95]. Different strains of influenza seem to have widely varying susceptibilities to SP-D and MBL, and at least part of this may be related to the number, location, and composition of glycans on HA. As noted above, reduced morbidity, mortality, and viral lung titers in mice and ferrets were observed with A(H1N1) and A(H3N2) IAV as the level of HA glycosylation increased [96–100]. Viruses that have low levels of glycosylation, such as the pandemic 1918 A(H1N1), 1957 A(H2N2), 1968 A(H3N2), and 2009 A(H1N1)pdm09 strains [96,98,101–106] and the zoonotic A(H5N1) [94,107–109] and A(H7N9) [110] strains, are relatively resistant to neutralization by SP-D and MBL in animal models, while highly glycosylated viruses are typically more susceptible [98,111–113].

Studies in mice show a rise in SP-D level in airways during influenza infection with either A(H1N1) or A(H3N2) IAV [96]. SP-D knockout mice show enhanced viral replication, lung inflammation, and morbidity after influenza infection [98,100–115], while expression of SP-D variants corrected the defects in A(H3N2) virus clearance and inflammatory responses [97]. However, mice are not natural hosts for influenza. Ferrets are considered to be a better model for human influenza infection [116], but the role of SP-D in the ferret model of influenza infection is less clear. Although SP-D appears to reduce replication of highly glycosylated A(H3N2) virus in ferrets [104,111], it may not be a major factor in this model [105].

In mice, MBL was present in bronchoalveolar lavage fluid by day 3 after infection with the mouse-adapted A(H1N1) strain A/Puerto Rico/8/1934 (PR8) [96]. MBL-knockout mice showed increased susceptibility to infection with a PR8 reassortant expressing the NA and NP segments and the highly glycosylated HA from the A(H3N2) strain A/Philippines/82 [111], but infection of MBL-deficient mice with A(H1N1)pdm09 or avian A(H9N2) strains, which are less heavily glycosylated, resulted in reduced severity and inflammation than in wild-type mice [117].

In humans, evidence that collectins protect against influenza is mainly indirect. Decreased SP-D expression in human lungs has been associated with fatal A(H5N1) influenza cases [108]. SP-D deficient groups such as smokers and people with chronic obstructive pulmonary disease, cystic fibrosis, and diabetes often suffer from more severe seasonal influenza disease [118–121]. Reduced serum levels of MBL in children was correlated with enhanced severity of A(H1N1)pdm09 infection [122].

Collectins protect against influenza infection by several mechanisms. Direct binding to mannose residues on IAV HA [123,124] and NA [82] blocks virus–receptor interactions and entry and leads to aggregation of viral particles. As well, formation of collectin–virus complexes enhances phagocytosis and modulates inflammatory responses [82,101,123–127]. Collectins inhibit influenza virus NA activity not only through direct binding to glycoprotein, but also through steric hindrance of the NA active site by lectins bound to glycans on HA [82,96,123].

While SP-D is generally anti-inflammatory [128,129], MBL is mainly a pro-inflammatory protein. After binding to pathogens, MBL can activate the lectin complement pathway via MBL-associated serine proteases, leading to complement-mediated lysis and/or opsonophagocytosis [130,131]. MBL may therefore act through both complement-dependent and independent mechanisms to neutralize influenza virus [111,132].

Glycosylation on specific residues of HA is associated with increased susceptibility to SP-D for some A(H3N2) and A(H1N1) IAV strains. The presence of an NLG site at residue 181 [99,101,107,115] or 262 [99] on the globular head of HA appears to be important for efficient neutralization of A(H3N2) IAV by SP-D, while glycosylation on residues 104, 142, and 293 may be important for A(H1N1) [101,107,133,134]. The importance of particular glycan sites for collectin sensitivity seems to depend on the extent of glycosylation. For example, A(H3N2) from the 1968 pandemic, which has lower levels of glycosylation, and current seasonal strains both contain an NLG site at residue 181. However, the 1968 strain is relatively resistant to both SP-D and MBL, while contemporary strains are sensitive [96,98].

Interactions between SP-D and its target glycans on viruses are influenced by glycan composition. Factors affecting the composition of glycans on influenza glycoproteins are not well understood, but may also be important for lectin-based inhibition [135,136].

#### Endocytic C-type lectins

Transmembrane C-type lectin receptors (CLRs) that recognize glycans of influenza virus HA and act as attachment/entry receptors are primarily expressed on alveolar and recruited macrophages, resident DCs, and B cells [20,22,23,137]. The importance of macrophages and DCs in host defense against IAV infection is well established [138,139]. Although it was thought that neither macrophages nor DCs can be productively infected with influenza virus, recent studies have shown productive infection of macrophages and DCs with A(H5N1) HPAI as well as A(H1N1) and A(H3N2) [20,140–143]. While seasonal influenza infection stimulates macrophage and DC chemokine and cytokine responses, leading to augmented immune responses, productive virus replication in these cell types may alter antiviral functions and enhance disease severity [144].

Transmembrane CLRs on macrophages include receptors that can recognize mannose, fucose, and *N*-acetyl-D-glycosamine (macrophage mannose receptor; MMR) or galactose (macrophage galactose-type lectin; MGL) on IAV [20,22]. As well, both macrophages and DCs have a receptor dendritic cell-specific ICAM3-grabbing integrin (DC-SIGN) for fucosylated and high-mannose oligosaccharides [23]. Alternative splicing generates several DC-SIGN isoforms, including both membrane-bound and soluble forms [145]. The role of soluble DC-SIGN in immune responses is not well understood. MMR and MGL are type I transmembrane proteins with several CRDs or CRD-like domains, and DC-SIGN is a type II membrane protein with a short amino terminal cytoplasmic tail, a neck region, and a single CRD.

Several studies have confirmed calcium-dependent lectin-mediated interactions between influenza and macrophages [20,22,25] and DC [21,23]. Such interactions could lead to infection of macrophages and DC, activation of these cell types, or both. While there is no direct evidence that CLR interactions with influenza are important for macrophage and DC activation, binding to endocytic CLR enhanced IAV infection of these cells [20–23,25]. For example, MMR or MGL on macrophages was shown to be principal co-receptors (along with SA-containing receptors) for A(H1N1) and A(H3N2) virus entry and infection [20,21,25]. DC-SIGN serves as an endocytic receptor for entry and infection with A(H1N1)pdm09 [138] and A(H3N2) IAV [146], and enhanced expression of DC-SIGN in activated B cells increases their susceptibility to HPAI A(H5N1) infection [147].

Virus uptake through CLRs depends on the level of influenza virus HA (but not NA) glycosylation [22]. Increased HA glycosylation, and glycosylation at specific positions, resulted in a higher level of influenza infection in both macrophages [20] and DCs [23,24,133]. Interaction between CLRs and glycans on HA may represent an important route for infection of macrophages and DCs immune cells with influenza viruses.

#### Galectins

The galectins constitute a family of soluble, primarily mammalian, C-type lectins that are produced by various tissues, including airway epithelium and immune cells [148]. They are typically between 14 and 35 kDa and contain a conserved CRD of about 130 amino acids that exclusively bind β-galactosides (such as lactose [Galβ(1–4)Glc] and N-acetyllactosamine [Galβ(1–4)GlcNAc]) [148] of influenza virus HA. Currently, 15 galectins have been identified in mammals, although only galectins -1 through -10, -12 and -13 (Gal -1 – Gal-10, Gal-12, and Gal-13) are found in humans [149]. They have a wide range of functions including mediation of cell–cell interactions, cell–matrix adhesion, and transmembrane signaling.

The ability of galectins to bind terminal galactose residues on the surface of viruses (including influenza) and act as PRRs has been recently recognized [150,151]. The effect of galectins on influenza disease seems to depend on the type of galectin and on genetic variation as well as the virus strain. Gal-1 has been shown to inhibit A(H1N1), A(H2N2), A(H3N2), A(H5N2), A(H6N1), and A(H7N9) IAVs [151–153], while Gal-3 enhanced A(H5N1)-induced pulmonary inflammation [154] and Gal-9 reduced immune responses to both A(H1N1) and A(H3N2) IAVs [155]. Genetic variants of human Gal-1 that encode higher lectin expression conferred more protection against A(H7N9) IAV infection [153]. Intranasal treatment with Gal-1 significantly enhanced survival of mice challenged with a lethal dose of A(H1N1) IAV, and GAL-1-knockout mice were more susceptible to infection. Enhancement of galectin expression by IAV–infection was reported for both humans (Gal-9) [156] and mice (Gal-1 and Gal-3) [152–154].

Interactions between IAV and galectins may also promote disease under some conditions. Human Gal-1 and -8 promote IAV adhesion to the surface of the target-cell without affecting the internalization stage [157], and binding of A(H1N1) IAV to Gal-1 and -3 enhanced virus adhesion to and productive infection of airway epithelial cells, and increased galectin-mediated adhesion of *S. pneumoniae* [158], suggesting a possible role for galectins in secondary bacterial infections following influenza.

#### Ficolins

Ficolins are a family of oligomeric soluble lectins with subunits consisting of CLD and fibrinogen-like domain (FBG) that are capable of interacting with glycans (including N-acetylglucosamine, fucose etc., but not mannose) on a variety of pathogens in both calcium-dependent and -independent ways [159]. Like MBL, ficolins initiate lectin-mediated complement activation. In humans, three types of ficolins (L, M, and H) have been described [160–163]. Ficolins L and H are mainly expressed by liver hepatocytes, while ficolin M is expressed by lungs, monocytes, and spleen [164–167]. H-ficolin is also expressed by type II alveolar and bronchial epithelial cells [167–170]. H-ficolin in human serum and BALF, and a purified recombinant variant, were shown to bind to IAV, block its HA activity, and inhibit replication of pandemic and seasonal IAVs in cell cultures [171]. Other studies have shown that H-ficolin and M-ficolin can inhibit replication of seasonal IAV in human monocytes [171,172]. Human L-ficolin and an L-ficolin-like molecule in mice were shown to bind HA and NA and inhibit IAV-infection both in tissue culture cells and mice [173].

## Concluding remarks

Lectins are ancient parts of the innate immune system that can bind glycans on viral glycoproteins, leading to direct viral neutralization and to priming the immune system to enhance viral clearance. Unlike most antibodies, lectins can bind to a wide range of influenza strains, making them attractive candidates for antiviral therapies.

For example, engineered human SP-D enhanced IAV binding and clearance and murine survival *in vivo* [174]. A recombinant fragment of human SP-D, containing only neck and CRD regions, showed ability to act as virus entry inhibitor as well as ability to down-regulate excessive virus-induced pro-inflammatory responses [175]. Recombinant porcine SP-D (either as the intact protein or as a fragment containing the neck and CRD only) inhibited IAV and IBV infection in epithelial cells of human trachea [94] and A(H3N2) IAV in *ex vivo* cultures of respiratory tract tissue [176,177], and was more effective than recombinant human SP-D.

MBL has also exhibited therapeutic potential with recombinant chimeric versions inhibiting IAV both *in vitro* (through complement activation and induction of viral aggregation [178]) and in mice (in part, by balancing cytokine and inflammatory responses [179]).

Despite the progress achieved in the field of influenza innate immunity in recent years, our understanding of the interactions between lectin, influenza, and host dynamics is incomplete. A better understanding of these relationships could lead to novel therapeutic and immunomodulatory approaches for the treatment of influenza disease.

## Abbreviations

CLD: collagen-like domain
CLR: C-type lectin receptor
ER: endoplasmic reticulum
FBG: fibrinogen-like domain
HA: hemagglutinin
IAV: influenza A virus
IBV: influenza B virus
ICV: influenza C virus
MBL: mannose-binding lectin
MGL: macrophage galactose-type lectin
MMR: macrophage mannose receptor
NA: neuraminidase
NLG: N-linked glycosylation
PAMP: pathogen-associated molecular property
PRR: pattern recognition receptor
RT: respiratory tract
SP-D: surfactant protein D
